# Identifying Candidate Genes that Underlie Cellular pH Sensitivity in Serotonin Neurons Using Transcriptomics: A Potential Role for Kir5.1 Channels

**DOI:** 10.3389/fncel.2017.00034

**Published:** 2017-02-21

**Authors:** Madeleine M. Puissant, Gary C. Mouradian, Pengyuan Liu, Matthew R. Hodges

**Affiliations:** ^1^Department of Physiology, Medical College of Wisconsin, MilwaukeeWI, USA; ^2^Neuroscience Research Center, Medical College of Wisconsin, MilwaukeeWI, USA; ^3^Center for Systems Molecular Medicine, Medical College of Wisconsin, MilwaukeeWI, USA; ^4^Cancer Research Center, Medical College of Wisconsin, MilwaukeeWI, USA

**Keywords:** RNA sequencing, serotonin, control of breathing, chemoreception, potassium channels

## Abstract

Ventilation is continuously adjusted by a neural network to maintain blood gases and pH. Acute CO_2_ and/or pH regulation requires neural feedback from brainstem cells that encode CO_2_/pH to modulate ventilation, including but not limited to brainstem serotonin (5-HT) neurons. Brainstem 5-HT neurons modulate ventilation and are stimulated by hypercapnic acidosis, the sensitivity of which increases with increasing postnatal age. The proper function of brainstem 5-HT neurons, particularly during post-natal development is critical given that multiple abnormalities in the 5-HT system have been identified in victims of Sudden Infant Death Syndrome. Here, we tested the hypothesis that there are age-dependent increases in expression of pH-sensitive ion channels in brainstem 5-HT neurons, which may underlie their cellular CO_2_/pH sensitivity. Midline raphe neurons were acutely dissociated from neonatal and mature transgenic SS^ePet-eGFP^ rats [which have enhanced green fluorescent protein (eGFP) expression in all 5-HT neurons] and sorted with fluorescence-activated cell sorting (FACS) into 5-HT-enriched and non-5-HT cell pools for subsequent RNA extraction, cDNA library preparation and RNA sequencing. Overlapping differential expression analyses pointed to age-dependent shifts in multiple ion channels, including but not limited to the pH-sensitive potassium ion (K^+^) channel genes *kcnj10* (Kir4.1), *kcnj16* (Kir5.1), *kcnk1* (TWIK-1), *kcnk3* (TASK-1) and *kcnk9* (TASK-3). Intracellular contents isolated from single adult eGFP^+^ 5-HT neurons confirmed gene expression of Kir4.1, Kir5.1 and other K^+^ channels, but also showed heterogeneity in the expression of multiple genes. 5-HT neuron-enriched cell pools from selected post-natal ages showed increases in Kir4.1, Kir5.1, and TWIK-1, fitting with age-dependent increases in Kir4.1 and Kir5.1 protein expression in raphe tissue samples. Immunofluorescence imaging confirmed Kir5.1 protein was co-localized to brainstem neurons and glia including 5-HT neurons as expected. However, Kir4.1 protein expression was restricted to glia, suggesting that it may not contribute to 5-HT neuron pH sensitivity. Although there are caveats to this approach, the data suggest that pH-sensitive Kir5.1 channels may underlie cellular CO_2_/pH chemosensitivity in brainstem 5-HT neurons.

## Introduction

Chronic physiological regulation of arterial pH is controlled by the kidney, but arterial oxygen, carbon dioxide (CO_2_), and pH levels are acutely regulated by chemoreflex-mediated changes in breathing. Central (brain) respiratory chemoreceptors are resident CNS cells (neurons/glia) whose pH- or CO_2_-dependent activity can affect ventilation (Richerson et al., 2005). Some brainstem cells have been identified as CO_2_/pH-sensitive and involved in the control of breathing, and in some cases the location, neurochemical identity, and molecular mechanism(s) of pH sensitivity have been determined. For example, accumulating data has demonstrated that CO_2_/pH-sensitive glutamatergic (Phox2b^+^) neurons in the retrotrapezoid nuclei (RTN) owe their intrinsic pH sensitivity to the combined expression of a specific Twik-related acid-sensitive potassium (K^+^) ion channel (TASK-2), and a novel G protein-coupled receptor (GPR4) (Kumar et al., 2015). The activity of Phox2b^+^ RTN neurons is also modulated by raphe-derived neuromodulators (serotonin and substance P), and *via* ATP released by local pH-sensitive glia (Mulkey et al., 2007a), which appear to rely on heteromeric, inwardly rectifying, pH-sensitive K^+^ (Kir4.1/Kir5.1) channels for their chemosensitivity (Wenker et al., 2010). Other populations of CO_2_-sensitive neurons in the Locus Coeruleus (LC; catecholaminergic neurons) and solitary complex are also thought to rely on yet to be identified pH-sensitive K^+^ channels (Martino and Putnam, 2007; Li and Putnam, 2013).

Serotonin-producing (5-HT) raphe neurons have also been shown to be pH- and CO_2_-sensitive, directly responding to hypercapnic and/or metabolic acidosis with increases in action potential firing rates in a variety of conditions and experimental preparations including behaving animals (Veasey et al., 1995, 1997; Wang et al., 2001; Bradley et al., 2002; Washburn et al., 2002; Iceman et al., 2013). The cellular response of 5-HT neurons to hypercapnic acidosis increases in magnitude on or after post-natal (P) day 12 in rats *in vitro* (Wang and Richerson, 1999; Wu et al., 2008). Specific subpopulations of 5-HT neurons in the rostral medullary raphe, which arise from the Erg2-expressing developmental cell lineage display an intrinsic pH sensitivity, and selectively innervate populations of brainstem neurons in respiratory-related nuclei (Brust et al., 2014). A modest pH sensitivity of young (<10 days of age) 5-HT neurons may depend upon their expression of pH-sensitive leak K^+^ channels such as TASK-1 and TASK-3 channels, although these channels do not appear to contribute to the CO_2_ chemoreflex in mature mice (Washburn et al., 2002; Mulkey et al., 2007b). Thus, the molecular determinants of the mature CO_2_/pH response in brainstem 5-HT neurons remain unclear (Massey et al., 2014; Teran et al., 2014).

Failure of mechanisms involved in cardiorespiratory control, and specifically dysfunction in the brainstem 5-HT system and the CO_2_ chemoreflex are believed to contribute to the pathophysiology of Sudden Infant Death Syndrome (SIDS; Panigrahy et al., 2000; Kinney et al., 2003, 2009; Paterson et al., 2006). SIDS is a leading cause of post-neonatal infant mortality in USA, and is thought to result from exogenous stressors applied during a critical period of development in an infant with an underlying abnormality (Filiano and Kinney, 1994). Several 5-HT system abnormalities have been identified in SIDS cases (Paterson et al., 2006; Duncan et al., 2010), and thus defining the developmental and regional shifts in gene expression profiles within brainstem 5-HT neurons and the potential genetic determinants of cellular CO_2_/pH chemosensitivity could provide key insights into the underlying biological dysfunction in SIDS.

Our goal here was to use age-related changes in gene expression profiles in groups of brainstem 5-HT neurons to identify developmentally regulated genes that may encode putative pH-sensitive molecules, including ion channels. By combining fluorescence-activated cell sorting (FACS) of primary eGFP-expressing 5-HT neurons from an established transgenic rat (SS^ePet-eGFP^ rats; Katter et al., 2013) and subsequent mRNA sequencing and differential gene expression analyses, we identified several genes that may encode proteins that contribute to cellular pH sensitivity in 5-HT neurons. In particular, the gene and protein expression of pH-sensitive Kir4.1 and Kir5.1 channel subunits increased with age in 5-HT neuron-enriched cell pools and raphe tissues, respectively. However, mRNA and/or protein for other pH sensitive K^+^ channels (TASK-1 and TASK-3) decreased or did not change with increasing age in 5-HT cell pools/raphe tissues. Single cell qPCR in adult eGFP^+^ 5-HT neurons confirmed heterogeneity of gene expression of Kir4.1, Kir5.1, TASK-1, TASK-3, and other genes. Immunofluorescence staining of adult brainstems showed Kir5.1 protein co-localized with brainstem neurons (including 5-HT neurons) and astrocytes, but Kir4.1 protein expression was restricted to brainstem astrocytes. Our data suggest a potential novel role for Kir5.1 within the medullary raphe. Additionally, our data suggest transcriptome comparisons can provide candidate genes that contribute to cellular pH sensitivity, but also suggest that more specific methods such as single-cell transcriptomics and subsequent RNA sequencing could provide an improved methodology for this purpose.

## Materials and Methods

Transgenic Dahl SS (SSMcwi) rats carrying the T2/ePet-eGFP transgene (Katter et al., 2013), which directs enhanced green fluorescent protein (eGFP) expression to Pet-1-expressing neurons [(SS-TgTn(T2ePet-eGFP)A1Mcwi rats; RGD strain ID: 8661234], were bred and maintained in-house and used for this study (*n* = 66). These rats have eGFP expression restricted to all 5-HT neurons, and will be referred to as SS^ePet-eGFP^ hereafter. Additional wild type Dahl Salt-sensitive (SS/JrHsdMcwi; SS) rats, an inbred strain generated and maintained by the Medical College of Wisconsin were used as control animals for this study (*n* = 6). All rats were housed in the Biomedical Research Center and allowed access to chow (Teklad) and water *ad libitum*, and maintained on a 12:12 h light/dark cycle. All experimental protocols were approved by the Medical College of Wisconsin Institutional Animal Care and Use Committee prior to experimental use.

### Cell Dissociation and Fluorescent Assisted Cell Sorting (FACS)

Young (P0-P3) or mature (P42-60) SS^ePet-eGFP^ rats were anesthetized with isoflurane (20% in propylene glycol v/v), rapidly decapitated, whole brains removed and placed in ice-cold Hibernate A (Life Technologies, #A1247501) for surgical isolation of the medullary raphe similar to that previously described (Wang et al., 1998). Tissue “wedges” were sectioned into rostral and caudal halves, placed in fresh Hibernate A, minced into small pieces and incubated in ice cold 2 mL Hibernate A or 2 mL of Accutase (Millipore; 30 min), pelleted at 380 g (2 min), re-suspended in 3 mL of Hibernate A and mechanically dissociated with a large-diameter sterile polyethylene transfer pipet similar to methods previously described by others (Guez-Barber et al., 2012). Tissues were gently titrated three to five times, and 2 ml of supernatant was transferred to fresh Hibernate A, and the process repeated until dissociation was complete. Dissociated cells were pelleted at 480 g (3 min) and re-suspended in Hibernate A and filtered (40 μm) and 4′, 6-diamidino-2-phenylindole (DAPI) added as a dead/live indicator prior to cell sorting [FACS Aria IIIa (BD Biosciences)]. Collection gating was established based on neuronal health (DAPI), forward and side scatter (size and complexity, respectively), by negative control tissues obtained from naïve SS rats to determine the threshold for eGFP (488 nm wavelength), and control cell samples incubated with anti-NeuN (neuron-specific nuclear protein, Abcam #AB104225) to determine neurons from debris. All cells subjected to sorting into eGFP^+^ or eGFP^-^ pools were collected in Trizol (Life Technologies; 5000–8000 cells per pool), where tissues from three young rats were combined for dissociation per cell sort, and one adult rat was used for each cell sort.

### RNA Extraction and mRNA-Sequencing

Total RNA was extracted from all collection tubes using previously described methods (Chomczynski and Sacchi, 2006), and quantified and qualified using a spectrophotometer (NanoDrop 2000; Thermo Scientific). All RNA from adult (*n* = 3–4) or young (*n* = 9–12) rats was pooled to create each cDNA library, and we generated three cDNA libraries per age and brain region; Young Rostral eGFP^+^ (YRP), Young Rostral eGFP^-^ (YRM), Young Caudal eGFP^+^ (YCP), Young Caudal eGFP^-^ (YCM), Adult Rostral eGFP^+^ (ARP), Adult Rostral eGFP^-^ (ARM), Adult Caudal eGFP^+^ (ACP), and Adult Caudal eFGP^-^ (ACM). Libraries were constructed (Illumina TruSeq RNA Sample prep Kit v2), validated (DNA 1000 chip; Agilent 2100 bioanalyzer), and sequenced (Illumina HiSeq 2000) in-house. Transcript analyses were completed with a customized data analysis pipeline for read mapping and alignment, transcript construction and quantification, and statistical expression using Bowtie, Tophat v2, and Cufflinks as previously described (Liu et al., 2014).

All differentially expressed transcripts from a given comparison were further analyzed using Ingenuity Pathway Analysis software. We focused on differentially expressed transcripts between YRP vs. ARP, YCP vs. ACP, ACP vs. ACM, ARP vs. ARM, YCP vs. YCM, and YRP vs. YRM and performed “core analysis” for each which provided outputs including Top Canonical Pathways, Upstream Analysis, Diseases and Functions, Regulator Effects, and Networks. An in-depth analysis of all upregulated plasma membrane molecule transcripts reaching significance (*P* > 0.01) between each library comparison as well as a targeted comparison of all differentially expressed K^+^ channels was performed.

### qRT-PCR Validation

To validate the specificity of the cell sort and age-dependent changes in gene expression, we used quantitative real-time PCR on a separate set of FACS-isolated eGFP^+^ and eGFP^-^ cells to quantify expression of the 5-HT neuron-specific genes *Tph2* and *Slc6a4*, and a housekeeping gene (18s) as an internal control for normalization.

To validate differentially expressed transcripts generated through mRNA-sequencing, we used quantitative PCR on a separate set of FACS-isolated eGFP^+^ and eGFP^-^ cells from a separate six animals each for four different age groups [P0-1, P7-8, P19-20, and adult (>P42)] of SS^ePet-eGFP^ animals using primers to quantify multiple transcripts including *Kcnj10* (Kir4.1), *Kcnj16* (Kir5.1), *Kcnk3* (Task-1), *Kcnk9* (TASK-3), *Kcna2* (Kv1.2), and 18S (internal control). Total RNA was extracted from FACS-isolated cells with Trizol (described above). Samples were then quantified and qualified (Nanodrop), converted to cDNA (RevertAid First Strand cDNA Synthesis Kit, #K1622, Thermo Scientific) and assayed using SYBR Green (iTaq Universal SYBR Green Supermix, Bio-Rad) technology as previously described (Yao et al., 2005) in triplicate. Gene expression was calculated using the delta-delta *C*t method (ΔΔ*C*_T_) where 18s was the housekeeping control. ΔC_T_ of GFP^+^ cells was the experimental group. ΔC_T_ of GFP^-^ or P0 cells were the control groups. Data are expressed as log fold change. Primer and probe sequences are shown in **Supplementary Table 1**.

### Isolation of Single Cell Contents and qRT-PCR

To verify 5-HT neuronal expression of candidate genes derived from FACS-RNA Sequencing, single intracellular 5-HT neuronal contents were isolated and gene expression for *Kcnj10, Kcnj16, Kcnk1, Kcnk3, Kcnk5, Kcnk9*, and *Kcna2* was measured using a TaqMan chemistry based Single Cell-to-C_T_ kit (#4458236, Ambion). SS^ePet-eGFP^ rat pup (P18-23) brains were isolated and then dissected in ice cold oxygenated (95% O_2_/5% CO_2_) slice solution (220 mM sucrose, 10 mM MgCl_2_, 2.5 mM KCl, 0.2 mM CaCl_2_, 10 mM dextrose, 1.3 mM NaPO_4_, and 26 mM NaHCO_3_ at a pH of 7.4) and then coronally sectioned (300 μm) using a vibratome. Rostral raphe sections were incubated for 30 min in nine parts slice solution and one part Ringers solution (124 mM NaCl, 3 mM KCl, 2 mM MgCl_2_, 2 mM CaCl_2_, 10 mM dextrose, 1.3 mM NaH_2_PO_4_, and 26 mM NaHCO_3_) before being placed into an electrophysiology recording chamber (no recordings were made). Single 5-HT neurons were identified under fluorescent live imaging. 0.5 μm diameter sterile micropipettes were backfilled with <1 μl of a modified intracellular solution according to Cadwell et al., 2016 [123 mM potassium gluconate, 12 mM KCl, 10 mM HEPES, 0.2 mM EGTA, 4 mM MgATP, 0.3 mM NaGTP, 10 mM sodium phosphocreatine, 20 μg/mL glycogen, and 1 U/μl recombinant RNase Inhibitor (#2313A, Takara) with pH 7.25] and using a micromanipulator (P97; Sutter Instruments), were placed onto the surface of single 5-HT fluorescent neurons such that there was a visible deflection or dimple in the membrane (seal resistance was not measured). Back pressure was applied and intracellular contents were collected using pulses negative pressure, which were visually verified using live fluorescent imaging (Cool Snap Nikon camera and Nikon elements D). Micropipettes were gently pulled out of the tissue and the micropipette tips (∼1 cm) were placed into an Alconox detergent solution (20 mg/ml) for 1 min to remove potentially attached tissue debris. The micropipette tip was then placed in Ringers solution for 1 min to remove the detergent. Isolated contents were then expelled into Lysis/DNase buffer provided by the Single Cell-to-CT kit (9 μl of lysis buffer and 1 μl of DNase), incubated for 5 min followed by a 2-min incubation after 1 μl of Stop Solution was added. Each sample was stored at -20°C until all samples were collected and then processed according to the Single Cell-to-CT kit. We confirmed that washing the micropipette tip had no unwanted effects and actually improved the purity of our samples by comparing gene expression of 5-HT (*Tph2, Ddc*), neuronal (*Map2, Nefl*), and glial (*Gfap*, *Aqp4*, and *Sox10*) specific gene markers and *Kcnj10* and *Kcnj16* from a subset of samples washed with Ringers + Ringers (*n* = 5 cells) and with Alconox + Ringers (*n* = 6 cells). There was no difference in *C*t-values between washing methods. We collected another seven samples that we used to measure the same 5-HT, neuronal, and glial markers in addition to K^+^ channel genes (*Knj10, Kcnj16, Kcnk1, Kcnk3, Kcnk5, Kcnk9*, and *Kcna2*). To gauge the expression pattern of *Kcnj10* and *Kcnj16* across 5-HT neurons we pooled all cells together (*n* = 18) and determined if there was or was not expression of these genes. A sample of bulk tissue was obtained and RNA isolated using a kit (74034, Qiagen) in order to compare 5-HT, neuronal and glial gene expression patterns between bulk tissue and our single cell isolation approach. qRT-PCR was run on a Quant Studio 6 Flex (Life Technologies).

### Western Blots

Additional SS^ePet-eGFP^ rats (*n* = 32) were anesthetized with isoflurane (20% in propylene glycol v/v), decapitated, and whole brains removed and flash frozen (2-methybutane with dry ice) and coronally sliced in a stainless steel matrix (1 mm; -20°C) before tissue punches of the midline raphe were obtained using an 18 g blunted needle as previously described (Puissant et al., 2015). Thirty microliters of Laemmli buffer (250 mM TrisCL, pH 6.8, 40% glycol, 8% SDS, 8% bME) was added to each single tissue punch and homogenized by sonication. Criterion pre-cast gels (Bio-Rad; 10–20% Tris-HCl, 26 well) were prepared, wells washed, and SDS running buffer (10% Tris/glycol/SDS-tween in H_2_0) added to submerge the entire gel. Eight microliters of sample was added to each well; electrophoresis ran at 80 V for 10 min until the sample and ladder migrated into the gel. Then, voltage was changed to 120 V and continued until sufficient ladder band separation. Protein transfer onto nitrocellulose paper ran for 1 h at 100 V in 25 μM Tris/192 μM Glycol/20% methanol. Nitrocellulose paper was placed into containers containing TBS-tween (TBS-T) on a laboratory rocker for 1 h. After 1 h block in 5% normal horse serum in TBS-T, primary antibodies [1:100 concentration of Kir4.1 (Alomone Labs, #APC-035), Kir5.1 (Alomone Labs, #APC-123), TASK-1 (Alomone Labs, #APC-024); and TASK-3 (Santa Cruz Biotechnology, #sc-11320)] and block (0.1% BSA) were mixed into TBS-T and placed into pouches with the nitrocellulose paper and incubated overnight at 4°C. Nitrocellulose paper was washed (×3) with TBS-T, secondary antibodies were added (1:10,000) and incubated for 1 h and then washed with TBS-T (×3) followed by TBS (×1). Blots were developed with Pico Chemiluminescense (Thermo Scientific), imaged with a ChemiDoc Imaging system (Bio-Rad), and processed with Image Lab software.

### Immunofluorescence Staining, Imaging, and Analyses

Another group of adult male SS^ePet-eGFP^ rats (*n* = 2) were anesthetized, transcardially perfused/fixed and brainstems removed, cryoprotected in 30% sucrose in PBS before freezing and sectioning (10 μm) coronally from -2.0 to 3.0 mm from obex and affixed to glass slides. Sections were immersed in 0.4% Triton x-100 in PBS (10 min), blocked with horse serum (5% in 0.2% Triton in PBS; 1 h), and sequentially incubated 1–2 days in primary antibodies targeting: tryptophan hydroxylase (anti-TPH; 1:1000, mouse monoclonal, Sigma-Aldrich T0678), Kir4.1 (anti-Kir4.1; 1:1000, rabbit polyclonal, Alomone Labs APC-035), Kir5.1 (anti-Kir5.1; 1:1000, Alomone Labs APC-123), glial acidic fibrillary protein (GFAP; AB5804, EMD Millipore) and/or neuronal nuclei (NeuN; mouse monoclonal (1:2000); MAB377, EMD Millipore). Antibody specificity has been shown by the manufacture, and through experiments in which Kir4.1 and Kir5.1 primary antibodies were pre-incubated with the peptide antigens before applying them to the tissues, which completely prevented detection of the proteins (data not shown). Secondary antibodies for immunofluorescence visualization (1:500) targeting the host species for the primary antibodies (anti-mouse or –rabbit) and conjugated to Alexa Fluor (A)594 or A488 (Vector Labs). Slides were cover slipped and images (20×) obtained using standard epifluorescence microscopy as previously described (Puissant et al., 2015). Confocal images were obtained using a scanning multi-photon Leica SP5 with 63x oil immersion lens (HCX PL APO CS, 1.4 numerical aperture) zoomed to 2.5x scanning at 200Hz with 146 μm pinhole, with Ar and HeNe tunable lasers and detectors. Images were post-processed (brightness/contrast enhanced, mean filter = 2) using the Fiji platform in ImageJ (Schindelin et al., 2012).

Raw 8 or 16 bit monochrome images of the red and green channels were also processed for each combination of proteins labeled to determine co-localization in ImageJ by calculating Manders’ correlation coefficients. This was calculated by determining the fraction of pixels of protein A overlapping with protein B, and the reverse calculation to derive correlation coefficients where 1.0 is a perfect co-localization and 0 no co-localization.

## Results

### Enriching for eGFP-Expressing Medullary Raphe 5-HT Neurons with FACS

**FIGURE 1:**
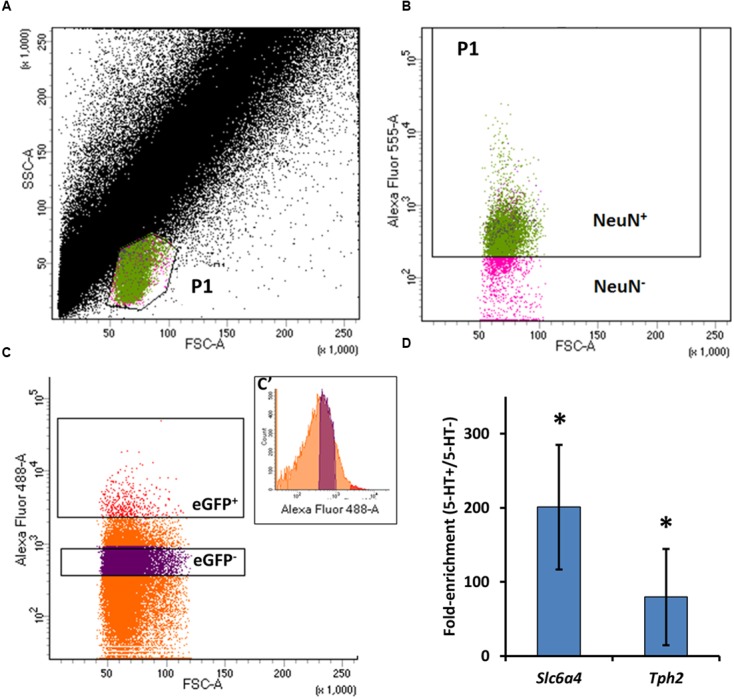
**Methods for FACS collection of dissociated eGFP-expressing (eGFP^+^) and non-eGFP (eGFP^-^) medullary raphe neurons. (A)** Representative data plotting forward-scatter (FSC) versus side-scatter (SSC) of all recorded sort events (cells). An area (P1) was identified as inclusive of raphe neurons, confirmed in **(B)** by sorting and plotting the FSC versus NeuN fluorescence of NeuN-labeled (Alexa Fluor 546-tagged) cells (Green events in **A** and **B**). **(C)** Gate establishment for the identification of eGFP^+^ (5-HT neurons) from ePet-eGFP:SS brainstem tissues by plotting FSC and the fluorescence intensity of Alexa Fluor 488 from collected P1/NeuN^+^ events, designated eGFP^+^ (red) or eGFP^-^ (purple). **(C’)** The distribution of Alexa Fluor 488 intensity versus event counts. **(D)** qPCR data showing fold-enrichment of 5-HT neuron-specific genes s*lc6a4* (SERT) and *tph2* (tryptophan hydroxylase 2) in eGFP^+^ (5-HT-enriched) versus non-5-HT neuron-enriched cell pools (*P* < 0.05; One-way ANOVA).

In order to derive candidate molecules underlying cellular CO_2_/pH sensitivity, we measured age-related changes and regional differences in gene expression in brainstem 5-HT neurons. We took advantage of an established rat line that carries an eGFP transgene under the control of an enhancer element for a 5-HT neuron-specific transcription factor Pet-1 (Katter et al., 2013). Brainstems from SS^ePet-eGFP^ rats were extracted, eGFP^+^ (5-HT) and eGFP^-^ (non-5-HT) neurons were dissociated then isolated into separate pools using FACS, similar to methods previously published (Guez-Barber et al., 2012). Conservative eGFP^+^ gating was established by sorting unlabeled medullary raphe cells from naïve SS rats, allowing for the identification of the putative neuronal population based on the resulting forward- and side-scatter data (**Figures 1A,B**). The scatterplot revealed a distinct cell population that corresponds to isolated neurons (Guez-Barber et al., 2012), which we confirmed in our sorted cell dissociates with or without fixation and conjugated primary antibodies specific for neurons (NeuN/Alexa 546; **Figure 1B**). This gated sub-population (P1) ranged from 68% to as high as 99.9% NeuN positive cells (data not shown), and from this collected pool we estimated that eGFP^+^ neurons represented 0.9–1.8% of these cells, supporting our assumption that we had established conservative gates for eGFP^+^ cells (**Figures 1C,C**’). Total RNA extracted from the FAC-sorted eGFP^+^ and eGFP^-^ cell pools was of high quality and sufficient quantity (Nanodrop 2000), and RT-qPCR experiments validated the specificity of the sort. We found 5-HT neuron specific genes (*Slc6a4* and *Tph2*), were significantly more expressed in eGFP^+^ (5-HT^+^) vs eGFP^-^ (5-HT^-^) cell pools of adult rats (**Figure 1D**), indicating significant 5-HT neuronal enrichment using this method.

### RNA Sequencing Reveals Large Developmental Shifts in Gene Expression in Medullary 5-HT Neurons

After validating our FACS enrichment strategy for brainstem 5-HT neurons, cDNA libraries for RNA Sequencing were prepared from FAC-sorted cell pools (20,000–70,000 cells/pool) collected from young (Y) or adult (A) rats, rostral (R) or caudal (C) raphe regions that were either eGFP-positive (P) or eGPF-negative (N; YRP, YRN, YCP, YCN, ARP, ARN, ACP, ACN). We noted that biological replicates within each experimental group yielded similar numbers of transcripts detected, and across all cDNA libraries there were high transcript yields (2.25–4.00 Gb), read number (23.1–41.0 million/library), mean quality score (94.2 or greater), and mapping rate (89.5% or better; data not shown). Libraries from common experimental groups were combined and differentially expressed genes determined across group comparisons using an adjusted *p*-value of *q* < 0.05 as the threshold for significance. We noted a far greater number of differentially expressed genes when making comparisons across ages (6234.3 ± 200.5 genes), and relatively few differences comparing cell types or regions within an age group (183.8 ± 44.8 genes). This major difference could reflect that these cells continue to develop after birth into adulthood, whereas the relatively small differences across cell type and region might suggest significant heterogeneity in gene expression profiles that reduce the statistical power to identify differential expression among all genes. Regardless, changes in gene expression patterns are greatly affected by age compared to cell type and raphe region (caudal vs. rostral).

### Candidate Molecules Underlying pH Sensitivity in 5-HT Neurons: Kir4.1 and Kir5.1

**FIGURE 2:**
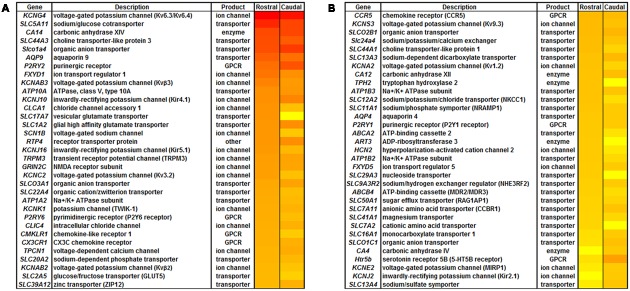
**All plasma membrane-associated genes that were upregulated with increasing age in 5-HT neuron-enriched cell pools.** Plasma Membrane molecules identified in IPA were significantly increased (*q* < 0.01) with increasing age in 5-HT neuron-enriched cell pools by FACS. **(A)** Gene name, description, protein type and relative change in expression (greatest increase (red) to smallest increase (yellow) for log fold-change with increasing age) is shown for identified plasma membrane-associated molecules. **(B)** Representation of the type of plasma membrane molecules categorized by transporters, ion channel, G-protein coupled receptors (GPCR), enzymes, or other.

The identification of several thousand differentially expressed genes in our main group comparisons (YRP vs. ARP, and YCP vs. ACP) necessitated an objective approach to identifying genes/molecules that change within increasing age. Given that cellular CO_2_/pH sensitivity in medullary 5-HT neurons is minimal immediately after birth but increases with postnatal age (Wang and Richerson, 1999; Wu et al., 2008; Hodges et al., 2009), and that CO_2_/pH sensitive 5-HT neurons are primarily located in the rostral medulla (Brust et al., 2014), we curated genes encoding membrane-associated ion transporters and/or channels that increase in expression with increasing age. Restricting the analysis to genes that were significantly upregulated (*q* < 0.01) with age in 5-HT neuron-enriched cell pools and designated to be “membrane-associated” by IPA yielded 66 genes (**Figure 2**). The top five plasma membrane genes upregulated with age which also had higher expression in rostral raphe cell pools were *Kcng4* (Kv6.4), *Slc5a11* (sodium/inositol co-transporter), *Ca14* (carbonic anhydrase), *Slc44a3* (solute carrier), and *Slco1a4* (solute carrier organic anion transporter). Of the 66 membrane-associated, differentially expressed genes, 50% (33 genes) encode small molecule/ion transporters, 30% (20) encode ion channels, 11% (7) encode GPCRs, 8% (5) encode enzymes (including Tph2) and <2% (1) other types of proteins. Of the ion channels genes identified, >50% (11 genes) encode known K^+^ channels, including (in order of greatest change in age-related expression) Kv6.3/Kv6.4, Kvβ3, Kir4.1, Kir5.1, Kv3.2, TWIK-1, Kvβ2, Kv9.3, Kv1.2, MIRP1, and Kir2.1.

**FIGURE 3:**
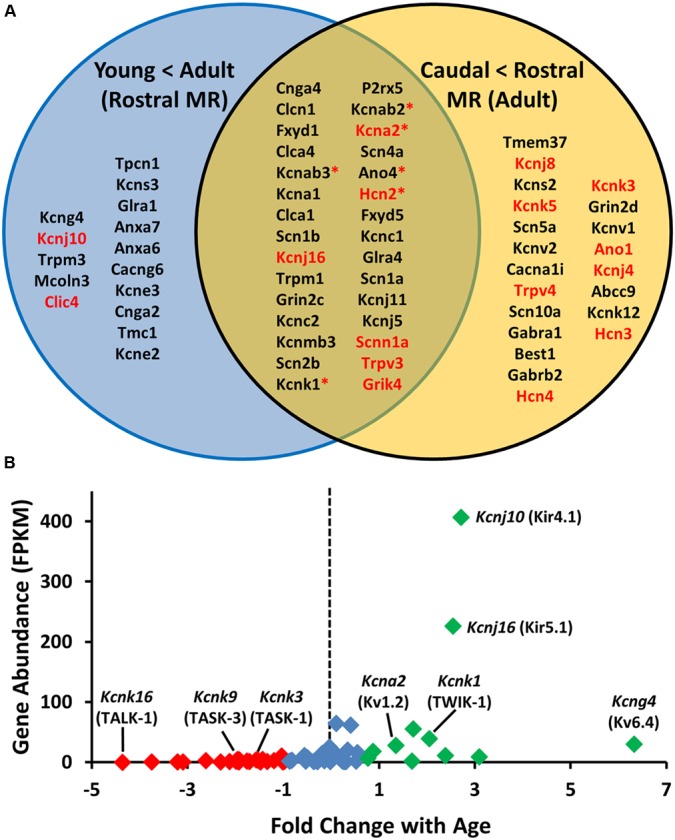
**Assumption-based identification of candidate genes through unique and overlapping differential expression patterns highlight the potential involvement of potassium ion (K^+^) channels. (A)** Upregulated plasma membrane molecules [>0.5 log-fold increase) from age-specific (young vs adult) rostral 5-HT RNA sequencing comparison; (blue)] and region-specific [caudal versus rostral adult 5-HT neurons; (yellow)] with common genes in middle (tan). Note that the genes listed in red encode proteins with known pH sensitivity, and those with asterisks bind to and/or associate with pH-sensitive proteins. **(B)** Fold change in expression (young vs. old) of all K^+^ channel genes plotted against overall abundance (fragments per kilobase of transcript per million reads; FPKM), where those in red are downregulated, green are upregulated, and those in blue are not differentially regulated with age.

Limiting the list of genes to all ion channel genes upregulated (<0.5-fold) across age within the rostral medullary raphe 5-HT neurons yielded a list 45 ion channels (blue circle; **Figure 3A**). Fifty-one genes were more highly expressed in rostral vs. caudal raphe 5-HT neuron-enriched cell pools from adult rats (yellow circle; **Figure 3A**), By comparison, we found 30 ion channel genes in mature 5-HT neurons that were upregulated (<0.5-fold change) in the rostral versus caudal region (**Figure 3A**). In general, most genes identified using this method were those encoding ion channel subunits, including 22 potassium channels, 9 chloride channels, 7 sodium channels, and 2 calcium channels. There were also multiple non-selective cation channels, including three hyperpolarization-activated, cyclic nucleotide-gated (HCN) channels, three transient receptor potential (Trp) channels, and a purinergic receptor subunit (*P2rx5*). Several of these differentially expressed channels have been implicated in cellular responses to pH changes in the brainstem, including *Kcnk1* (TWIK-1), *Kcnk3* (TASK-1), *Kcnk5* (TASK-2), *Kcnk9* (TASK-3), *Kcna2* (Kv1.2), *Kcnj4* (Kir2.3), *Trpv3* (TRPV3), *Kcnj10* (Kir4.1), and *Kcnj16* (Kir5.1) (Washburn et al., 2002; Putnam et al., 2004; Riley et al., 2010; Wang et al., 2013).

Given the preponderance of K^+^ channels in the above comparisons and our assumption that the genetic determinants of cellular chemosensitivity will increase in expression with increasing age, we focused on all K^+^ channels found in 5-HT neuron-enriched cell pools and determined which of these channel genes were expressed more highly in 5-HT versus non-5-HT neuron cell pools (**Figure 3B**). Among the age-related changes in expression of all K^+^ channels detected in the rostral raphe 5-HT neurons, we found 25 genes were downregulated including TASK-1 and TASK-3 (red; **Figure 3B**), 13 were upregulated including TWIK-1, and Kir4.1 and Kir5.1 (green; **Figure 3B**), and 31 were unchanged with age. However, expressing these fold-changes in expression relative to their abundance in adult 5-HT neurons (**Figure 3B**) suggested that Kir4.1 and/or Kir5.1 may be ideal candidates to underlie pH sensitivity in 5-HT neurons as they were both increased with age and in much higher abundance relative to all other K^+^ channels expressed.

### Single Cell PCR Validation Confirms 5-HT Neuron Specific Expression of *Kcnj10* and *Kcnj16*

The RNA-Seq data showed significantly greater abundance (*q* < 0.05) of 5-HT neuron-specific genes including *Tph2*, *Ddc*, and S*lc6a4* (SERT) in eGFP^+^ cell pools versus eGFP^-^ pools from the rostral raphe of mature rats, confirming successful 5-HT enrichment. However, we did not measure significantly greater 5-HT neuron-specific genes in our other group comparisons. For unknown reasons, 5-HT neuron enrichment was not as robust in cells collected from the caudal raphe of young or mature rats, similar to other reports (Wylie et al., 2010). To our surprise, we also found multiple glial-specific genes in all 5-HT neuron-enriched cell pools, including *Gfap*, *Aqp4*, and *Sox10*. Thus, the sequencing data indicated our dissociation and FACS methods had enriched for 5-HT neurons but also likely included fragments of neighboring glial cells, which brought into question the 5-HT neuron-specific expression of our candidate genes.

**FIGURE 4:**
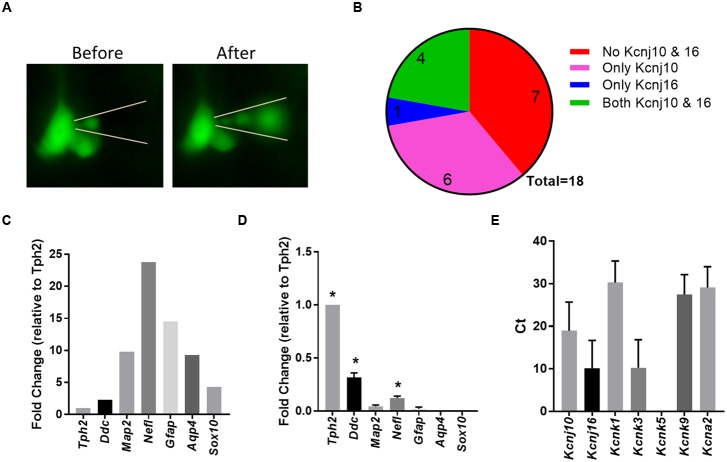
**Single cell qPCR from individual 5-HT neurons confirmed Kcnj10 and Kcnj16 gene expression. (A)** Representative visualization of eGFP-expressing brainstem 5-HT neuron and the isolation of the intracellular contents. **(B)** Number of single 5-HT neuron samples that did not express *Kcnj10* & *Kcnj16* (red), that only expressed *Kcnj10* (pink), only *Kcnj16* (blue), and both *Kcnj10* and *Kcnj16* (green). **(C)** Representative expression pattern of 5-HT specific (*Tph2* and *Ddc*), neuronal specific (*Map2* and *Nefl*), and glial specific (*Gfap, Aqp4*, and *Sox10*) gene markers from a bulk raphe tissue homogenate normalized to *Tph2* expression. **(D)** Expression pattern of 5-HT specific, neuronal specific, and glial specific genes among single 5-HT neurons normalized to *Tph2* demonstrating purity and specificity of single cell intracellular content isolation using single cell qRT-PCR (*n* = 7; ^∗^ vs. *Gfap*, *Aqp4*, and *Sox10* by One-Way ANOVA). **(E)** Single cell qRT-PCR *C*t-values demonstrating 5-HT specific expression of K^+^ channel genes, including *Kcnj10* and *Kcnj16* (*n* = 7).

Due to these caveats, it was necessary to validate or invalidate the expression of *Kcnj10*, *Kcnj16*, and other K^+^ ion channel genes specifically in adult 5-HT neurons. Micropipettes were used to isolate the intracellular contents of individual eGFP^+^ 5-HT neurons from acute medullary tissue slices from adult SS^ePet-eGFP^ rats (**Figure 4A**). Gene expression of glial-specific (G*fap*, *Aqp4*, and *Sox10)*, neuronal-specific (*Map2* and *Nefl)* and 5-HT neuron-specific (*Tph2*, *Ddc*) markers among individual 5-HT neurons was measured and normalized to *Tph2* to compare against a representative bulk raphe homogenate (**Figure 4C**). As expected, intracellular contents isolated from eGFP^+^ 5-HT neurons had significantly higher normalized expression of *Tph2*, *Ddc* and *Nefl* (*P* < 0.05; One-way ANOVA) compared to all three glial markers (*Gfap*, *Aqp4*, and *Sox10*; **Figure 4D**) as determined by single-cell qRT-PCR. Of the 18 eGFP^+^ 5-HT neurons assayed, 33% of the 5-HT neurons expressed only *Kcnj10* (6/18), 6% expressed solely *Kcnj16* (1/18), 22% expressed both (4/18) and 39% expressed neither gene (7/18; **Figure 4B**). In a subset of these cells, we also probed for relative expression of additional K^+^ channel genes (**Figure 4E**), where *C*_T_-values were lowest (higher gene expression) for *Kcnj16*, *Kcnk3* (TASK1), and *Kcnj10*. Higher *C*_T_-values (lower relative expression) were found for *Kcnk1* (TWIK-1), *Kcnk9* (TASK-3), *Kcna2* (Kv1.2), and as expected, we found no expression of *Kcnk5* (TASK-2; Gestreau et al., 2010; Wang et al., 2013). Therefore, although it is likely that our RNA Sequencing data reflected some degree of glial contamination, individual 5-HT neurons were confirmed to express one or both *Kcnj10* and *Kcnj16* mRNA.

### Developmental Increases in Kcnj10/Kir4.1 and Kcnj16/Kir5.1 in 5-HT Neuron-Enriched Cell Pools

**FIGURE 5:**
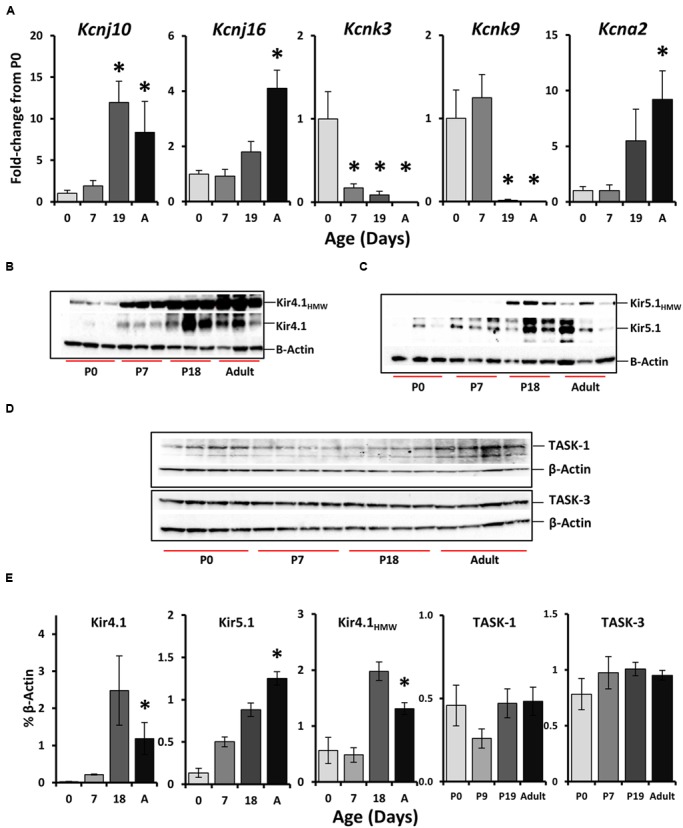
**Age-related changes in the expression of select K^+^ channels the rostral medullary raphe. (A)** *Kcnj10, Kcnj16, Kcnk3, Kcnk9*, and *Kcna2* gene expression patterns across age (P0, 7, 19, and adult) within the rostral raphe (^∗^*P* < 0.05 vs. P0 by one-Way ANOVA). Western blots from raphe tissues collected from different ages showed increases in both a high molecular weight band (∼250 kDa) Kir4.1_HMW_ and expected (42.5 kDa) Kir4.1 bands **(B)**, Kir5.1_HMW_ (∼250 kDa) and expected (48 kDa) Kir5.1 bands **(C)**, and TASK-1 (45–65 kDa) and TASK-3 (45 kDa) **(D)** with respective quantification normalized to β-actin (**E**; ^∗^*P* < 0.05 vs. P0 by one-way ANOVA). Note that the quantification for Kir5.1 protein in **(E)** was from two blots from P0 (*n* = 3), P7 (*n* = 4), P18 (*n* = 6), and adult (*n* = 3), but only one is shown in **(C)**.

Chemosensitivity increases with increasing age in 5-HT neurons *in vitro*, and thus we hypothesized that candidate genes that underlie cellular chemosensitivity may also increase in expression with increasing age. To better define the temporal expression pattern of the identified K^+^ channels, we performed additional FACS experiments in SS^ePet-eGFP^ rats at 0, 7–8, 18–19, and 42–60 days of age. PCR amplification of selected genes in the 5-HT neuron-enriched cell pools was normalized to 18s and expressed as a fold-change (ΔΔ*C*_T_) from P0 values (**Figure 5A**). There were significant increases in expression of *kcnj10*, *kcnj16*, and *kcna2* in 5-HT neuron-enriched cell pools with increasing age (**Figure 5A**; *P* < 0.05; One-way ANOVA), where expression levels were increased by P19 or after. In contrast, mRNA expression levels of both *kcnk3* and *kcnk9* significantly decreased with increasing age in 5-HT neuron-enriched cell pools (**Figure 5**; *P* < 0.05; One-way ANOVA). These PCR data differ somewhat from the single cell PCR data from individual adult 5-HT neurons (**Figure 4E**), where there was robust gene expression of *Kcnk3* and *Kcnk9*, which is more consistent with previous reports (Washburn et al., 2002).

Western blot quantification of protein levels of Kir4.1 and Kir5.1, but not TASK-1 and TASK-3 in bulk raphe tissue punches also showed developmental changes in expression (**Figures 5B–E**). Western blots probing for Kir4.1 (**Figure 5B**), Kir5.1 (**Figure 5C**), or TASK-1 and TASK-3 (**Figure 5D**) in medullary raphe tissues samples from rats at P0 (*n* = 3), P7 (*n* = 4), P18 (*n* = 6), or adult (*n* = 3) ages (P49–60) for each antibody showed distinct bands at the expected molecular weights. Kir4.1 (∼42.5 kDa) and Kir5.1 (∼48 kDa) blots both showed a single-high molecular weight (HMW) band common to both blots around the same molecular weight (∼250 kD; **Figures 5B,C**), which we speculate may represent a heteromeric Kir4.1/5.1 channel or homotetrameric channels. Quantification of these individual or common protein bands relative to β-actin showed significant increases in expression with increasing age relative to P0 (**Figure 5E**; *P* < 0.05; One-way ANOVA), including increases in Kir4.1, Kir5.1 and the putative heteromeric channel. In contrast, there were no age-related changes in protein expression for both TASK-1 and TASK-3 in western blots from additional tissues obtained at similar ages (**Figure 5E**; *P* > 0.05; One-way ANOVA). Thus, Kir4.1 and Kir5.1 gene and protein expression increase significantly with increasing age in 5-HT neuron-enriched cell pools and raphe tissues, whereas TASK-1 and TASK-3 either decrease (mRNA) or do not change (protein) with age.

### Localization of Kir4.1 and Kir5.1 Channel Proteins in the Medullary Raphe

**FIGURE 6:**
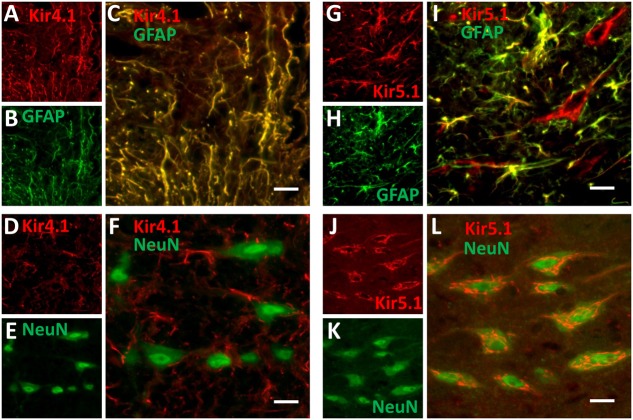
**Kir4.1 and Kir5.1 expression in the medullary raphe.** Immunofluorescence of fixed-frozen brainstem sections labeled with primary antibodies targeting Kir4.1 **(A–F)** or Kir5.1 **(G–L)** along with neuronal (NeuN) and astrocytic (GFAP) markers. Kir4.1 is expressed in medullary raphe astrocytes **(A–C)**, but not neurons **(D–F)**. Kir5.1 is expressed in medullary raphe astrocytes **(G–I)** and neurons **(J–L)**. The correlation coefficients for co-localization were as follows: **C** = 0.926, **F** = -0.063, **I** = 0.888, and **L** = 0.608. Images were obtained at 20×, and scale bars = 20 μm.

**FIGURE 7:**
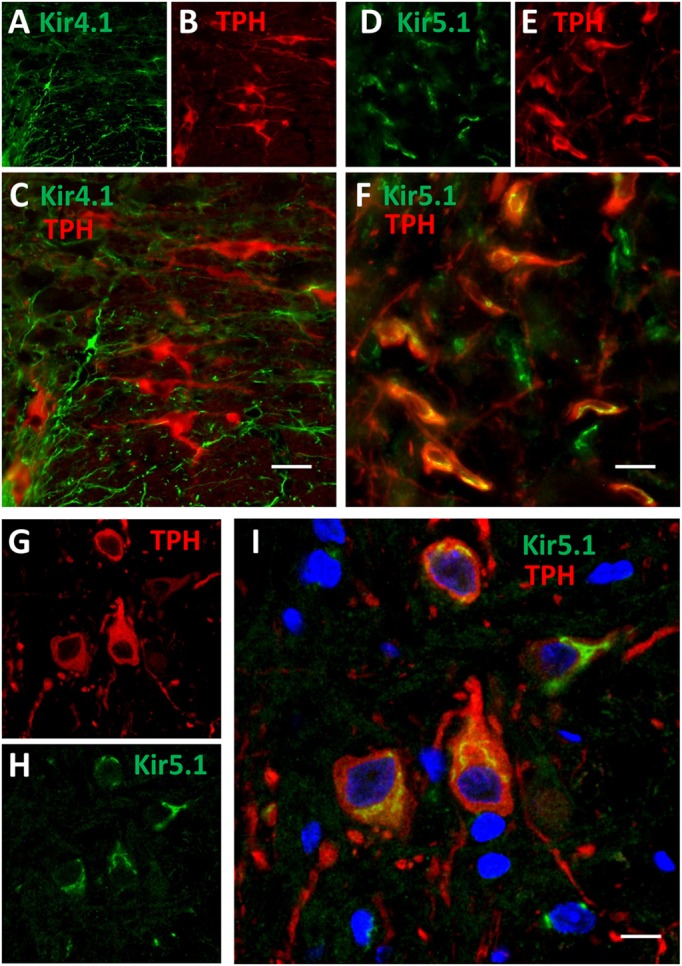
**Kir5.1 but not Kir4.1 protein is co-localized to brainstem 5-HT neurons.** Epifluorescence images of fixed-frozen brainstem sections labeled with primary antibodies targeting Kir4.1 (green; **A**) and TPH (red; **B**) or Kir5.1 (green; **D**) and TPH (red; **E**). Overlay images of indicate that Kir5.1 (correlation coefficient = 0.605; **F**) but not Kir4.1 (correlation coefficient = 0.109; **C**) is co-localized to TPH^+^ 5-HT neurons (yellow), where TPH and Kir5.1 co-localization appears primarily perinuclear and to a lesser extent along the plasma membrane **(G–I)**. Confocal images (63×, 2.5 magnification) of brainstem tissues double-labeled with TPH (red; **G**) and Kir5.1 (green; **H**) also show intense co-localization (yellow; **I**) along the perinuclear and to a lesser extent plasma membrane regions. Scale bar = 20 μm **(C,F)** or 10 μm **(I)**.

Kir4.1 and Kir5.1 channel subunits are largely thought to be co-expressed in glial cells throughout the CNS, although there is some evidence for Kir5.1 channel expression in neurons. **Figure 6** shows images of immunofluorescence of fixed-frozen brainstem sections labeled with primary antibodies targeting Kir4.1 (**Figures 6A–F**) or Kir5.1 (**Figures 6G–L**) along with neuronal (NeuN) and astrocytic (GFAP) markers. Kir4.1 expression in the medullary raphe (and throughout the brainstem) was restricted to GFAP-expression astrocytes (**Figures 6A–C**), and was not found co-localized with the neuronal marker NeuN (**Figures 6D–F**). Kir5.1 protein was also found co-localized with the vast majority of GFAP-expression astrocytes in the medullary raphe (**Figures 6G–I**), but was also found in cells other than glia. Kir5.1 expression was co-localized to many neurons in the brainstem, and in particular appeared to be co-localized mostly with neurons in the rostral medullary raphe (**Figures 6J–L**). Consistent with these observations, there was no observable Kir4.1 protein that co-localized with 5-HT neurons (**Figures 7A–C**), but we found co-localization of Kir5.1 with 5-HT neurons in the medullary raphe (**Figures 7D–F**), where the 5-HT neuron-specific expression of Kir5.1 appeared to be perinuclear and to a lesser extend membrane bound (**Figures 7G–I**). These data demonstrate the presence of these Kir isoforms within the raphe, but suggest only Kir5.1 protein was expressed in 5-HT (and other) neurons while Kir4.1 protein was localized to neighboring glia.

## Discussion

The molecular mechanisms conferring intrinsic CO_2_ and/or pH chemosensitivity of medullary raphe 5-HT neurons remain unknown. The ventilatory response to hypercapnia has generally been shown to increase throughout post-natal development in rodents and humans (Moss and Inman, 1989), and the intrinsic CO_2_/pH sensitivity of brainstem 5-HT neurons appear to also increase throughout post-natal development (Wang and Richerson, 1999; Wu et al., 2008). SIDS appears to result due to cardiorespiratory control failure (Thach, 2005), particularly during a critical window of development where SIDS rates are highest (Kinney and Thach, 2009). Understanding fundamental properties of 5-HT neurons, developmental aspects of their function, and their role in cardiorespiratory control mechanisms is critical to improving our understanding of SIDS given the identification of several 5-HT system abnormalities reported in SIDS cases (Paterson et al., 2006; Duncan et al., 2010).

Herein, we tested the hypothesis that gene transcripts encoding pH sensitive proteins, including known pH-sensitive ion channels are developmentally regulated in 5-HT neurons by applying fluorescent cell sorting and subsequent RNA sequencing to determine age-related and region-specific differential gene expression profiles. Further, we developed a robust technique to isolate the intracellular contents of individual adult 5-HT neurons to validate the mRNA expression of some of the candidate genes identified from RNA Sequencing. The data showed that adult brainstem 5-HT neurons express genes that encode pH sensitive molecules, including ion channels. Candidate genes were selected based on three major underlying assumptions, that: (1) genes encoding the CO_2_- and/or pH-sensitive proteins driving cellular chemosensitivity in 5-HT neurons increase in expression with increasing age (Wang and Richerson, 1999; Wang et al., 2001), (2) rostral raphe (magnus) regions contain significantly greater numbers of chemosensitive 5-HT neurons compared to caudal raphe (obscurus) regions (Brust et al., 2014), and (3) that the candidate genes would encode proteins associated with or embedded within the extracellular membrane. These assumptions gave rise to transcriptome comparisons from 5-HT neuron-enriched cell pools across age and raphe regions, allowing lists of >6000 developmentally regulated genes to be reduced to <100 candidate genes. Further restriction of the gene list to K^+^ ion channels alone, we found that of the ∼70 K^+^ channel genes expressed in 5-HT-enriched cell pools, there were a select few K^+^ channel genes that increased with age and were in high abundance; *Kcnj10* and *Kcnj16*. These genes were of particular interest as they encode the inwardly rectifying K^+^ channel subunits Kir4.1 and Kir5.1, respectively, which can form heteromeric “leak” K^+^ channel with pH-sensitivity well within the physiologic range (pKa ∼6.8 – 7.5; reviewed in Putnam et al., 2004). Gene expression of both *Kcnj10* and *Kcnj16* increased throughout postnatal age in 5-HT neuron-enriched cell pools with FACS, which agreed well with the developmental increases in protein expression of Kir4.1 and Kir5.1 measured in raphe tissues. Finally, qPCR from the intracellular extracts from individual adult eGFP^+^ 5-HT neurons also showed expression of *Kcnj10*, *Kcnj16* or both genes, further suggesting that these genes may encode pH-sensitive K^+^ channels that could underlie cellular pH sensitivity of chemosensitive brainstem 5-HT neurons.

Although Kir4.1/5.1 channels and others containing Kir4.1 or Kir5.1 subunit-containing channels have previously been postulated (Putnam et al., 2004; Wu et al., 2004) or demonstrated to contribute to cellular pH sensitivity (Wenker et al., 2010; D’Adamo et al., 2011) in the CNS, their expression has been considered to be mainly if not exclusively expressed in glial cells (reviewed in Hibino et al., 2010). However, there are reports demonstrating mRNAs for both Kir4.1 and Kir5.1 in brainstem neurons (Wu et al., 2004) and specifically in FAC-sorted pools of 5-HT neurons (Wylie et al., 2010) or in individual mouse 5-HT neurons (Okaty et al., 2015), consistent with our RNA sequencing and single cell PCR data. However, the immunofluorescence staining in adult rat brainstems showed that Kir4.1 protein expression appeared exclusively glial, with complete co-localization with GFAP expression without any observable co-localization with NeuN expression. In contrast, we found Kir5.1 expression co-localized with GFAP throughout the brainstem, but also found Kir5.1 expression in many brainstem neurons. Kir5.1 (but not Kir4.1) immunoreactivity was also co-localized with raphe 5-HT neurons, which further supports the nomination of *Kcnj16* (Kir5.1) subunit-containing channels as potential contributors to cellular pH-sensitivity in 5-HT neurons. Confocal microscopy indicated that the somatic sub-cellular localization for Kir5.1 was largely perinuclear and to a lesser extent distributed to the cell membrane, although this would need to be assessed with electron microscopy to be certain.

Additional functional evidence is needed for complete validation of the hypothesized role of Kir5.1 as pH sensors in 5-HT neurons, but there are data that suggest Kir5.1 channels contribute greatly to cellular pH responses in other chemosensitive neurons. Cellular responses to acidosis in catecholaminergic LC neurons rely on 4-AP-sensitive (A-type) currents implicating Kir channel involvement (Pineda and Aghajanian, 1997; Li and Putnam, 2013). Furthermore, D’Adamo et al. (2011) showed a reduced cellular responses to reductions in pHi upon removal of prepulses of NH_4_Cl in LC neurons in Kir5.1 knockout mice, and ventilatory data from Kir5.1 knockout mice show a blunted hypercapnic ventilatory response under physiologic conditions in mice challenged with 21% O_2_ (although not with hyperoxic hypercapnia) (Trapp et al., 2011), consistent with a role of Kir5.1 channels in the ventilatory CO_2_ chemoreflex (although this was not the ultimate conclusion of that study). Thus, Kir5.1 subunit-containing channels contribute to cellular pH responses in aminergic LC neurons and to the ventilatory CO_2_ chemoreflex *in vivo*, but functional contributions to pH sensitivity in 5-HT neurons remains unknown.

### Caveats and Considerations

There are several caveats and considerations with the current data, in particular the difficulties in determining the specificity of our cell sorting methods based on gene expression. We took advantage of established methods previously reported for sorting primary dissociated, antibody-tagged CNS cells (Guez-Barber et al., 2011; Guez-Barber et al., 2012) for our eGFP-based approach. Initial gating was based on cell viability (DAPI) and neuronal populations targeted by side-scatter selection confirmed by NeuN-tagged cell sorts. Further, conservative gates were set for eGFP inclusion in an effort to purify the collected 5-HT cell pools. However, the sequencing data suggest that for unknown reasons, the 5-HT neuron enrichment methods with FACS worked better for some conditions than others. In addition, we could readily find glial-specific genes in our 5-HT neuron-enriched cell pools, which could suggested that glial cells were likely included in our 5-HT neuron-enriched cell pools. This conclusion is, however, counterbalanced against the results of our single cell PCR data, which not only demonstrate a high degree of specificity for 5-HT neuron-specific gene expression (11 of 11 expressed *Tph2, Ddc, and Nefl*), they also show that many 5-HT neurons express mRNAs for proteins considered glial-specific, including *Gfap* and *Aqp4* (9 of 11) and *Sox10* (3 of 11). In addition, other groups have shown single cell transcriptome data from mature mouse 5-HT neurons, in which they found similar expression patterns of genes considered to be glial-specific (Okaty et al., 2015). Thus, the presence of “glial-specific” genes in the sequencing data do not necessarily speak to the efficacy of the cell sorting, but rather it speaks to a potential disconnect with measurements of gene expression and whether that translates to protein expression. Our data nominated both Kir4.1 and Kir5.1, but only one of these highly expressed genes gave rise to the corresponding protein in 5-HT neurons. There may be small amounts of Kir4.1 proteins being expressed, but it was not detectible with immunofluorescence.

Despite the inherent disconnect between gene and protein expression, the use of transcriptome comparisons to identify molecular determinants that distinguish between cellular properties or populations may still be useful as a discovery-based approach. For example, the molecular basis for infrared radiation detection in venomous pit vipers (TRPA1) was identified using transcriptome comparisons of trigeminal ganglia from rattlesnakes and other non-pit species. With regard to the molecular basis for chemosensitivity in brainstem 5-HT neurons, the approach may require more specificity than cell sorting of primary neurons can provide, in part due to known heterogeneity in this cell population and for the reasons stated above. Our single cell PCR data highlight this cell-to-cell variation in gene expression, which is also reflected in other single cell transcriptome data (Okaty et al., 2015). An improvement therefore would be to compare gene expression profiles among brainstem 5-HT neurons based on function (pH-sensitive vs. –insensitive for example) at the single cell or subpopulation level to enhance the identification of true positives. Finally, any approach using differential gene expression to identify the molecular basis of a function requires functional validation in addition to PCR and protein expression validation experiments. Thus, our gene candidate *Kcnj16* (Kir5.1) requires additional functional validation *in vitro* and *in vivo*.

Another consideration is that functional Kir5.1 channels are thought to require heteromeric assembly with other Kir subunits, such as Kir4.1, Kir4.2, or potentially Kir2.1 (Xu et al., 2000). This is largely based on the data showing that heterologous expression of Kir5.1 channels alone does not yield a functional channel (Pessia et al., 1996). If this is the case, the additional co-expressed subunit in 5-HT neurons, which would bind to Kir5.1 subunits and form a functional heteromeric channel is not clear, as we found: (1) no Kir4.1 protein expression in 5-HT neurons, (2) little or no Kir4.2 gene expression in the RNA sequencing data, and (3) no evidence of Kir4.2 protein in the brainstem with immunohistochemistry (data not shown). Kir2.1 gene expression has been localized to neurons, but we and others (Okaty et al., 2015) found little or no evidence of Kir2.1 gene expression in 5-HT neurons. Although, we cannot identify a specific binding partner that would lead to a functional Kir5.1-containing channel, the addition of this subunit may be required for a Kir channel to exhibit enhanced pH sensitivity with a pKa in the physiologic range. But, these observations also lead us to question if indeed homomeric Kir5.1 channels, under certain circumstances, could functionally contribute to resting membrane potential and/or alterations in membrane excitability. Functional homomeric Kir5.1 channels have been demonstrated when they are co-expressed with the post-synaptic density 95 (PSD-95) protein, which directs Kir5.1 channel expression to the plasma membrane (Tanemoto et al., 2002). This membrane localization is reversibly prevented by activation of protein kinase A, suggesting regulation of the Kir5.1 channel localization may modulate its function. Other data indicate functional Kir5.1 channel homomers in the retina do not require co-localization with Kir4.1, Kir4.2, or PSD-95 but may require an additional factor for independent functionality (Ishii et al., 2003). Our confocal imaging of brainstem 5-HT neurons suggested that most Kir5.1 channel protein was perinuclear with only a fraction at or near the plasma membrane, similar to previous descriptions (Tanemoto et al., 2002). Further investigation is required to determine if this sub-cellular localization is the “default” state of the channel, and only under certain circumstances is it localized to the plasma membrane to alter cellular function.

Lastly, we have chosen to focus on the validation of a small number of identified ion channels that were nominated by the RNA sequencing data, although there were many more genes identified. Our focus on K^+^ channels was based on the long-standing assumption that pH sensitive currents are most likely due to the closure of “leak” K^+^ channels (Putnam et al., 2004). However, equally plausible is the concept of the activation of a cation current by hypercapnic acidosis leading to depolarization of respiratory chemoreceptors, which has previously been hypothesized for chemosensitive brainstem 5-HT neurons (Richerson, 2004). Moreover, we further limited our analyses to genes that encode or modulate channels with known pH-sensitivity, where there could be other novel candidates that could be functionally altered through various mechanisms (alternative splicing, post-translational modifications, etc.), or others prominently expressed in other organs that are repurposed in neurons. For example, one of the developmentally regulated genes in our sequencing data is *Scnn1a*, which encodes the alpha-ENaC a sodium channel that regulates electrolyte transport across membranes particularly in the kidney. However, alpha-ENaC activity is highly pH-dependent, and was recently found to be co-localized to 5-HT neurons in the brainstem.

Finally, it is quite possible that cellular pH sensing mechanisms require multiple pH-sensitive ion channels/proteins, including complementary expression of pH-insensitive ion channels. This concept has a great deal of merit, particularly in light of the requirements of combined expression of two pH-sensitive proteins TASK-2 and Gpr4 for direct pH sensing in chemosensitive RTN neurons (Kumar et al., 2015), and other data showing Nalcn, KCNQ, HCN, THIK-1 and others altered chemosensory responses in RTN chemoreceptors independent of a role in direct pH sensing (Lazarenko et al., 2010; Hawryluk et al., 2012; Hawkins et al., 2015; Shi et al., 2016). Thus, additional changes in gene expression of pH-insensitive ion channels could also contribute to pH sensing mechanisms, but may not be identified using the approach herein. Despite the stated limitations, this cell-specific gene expression-based approach could be ideal to identify potential panels of pH sensitive proteins (and those that have indirect, supportive roles) by comparing multiple cell populations, which would be difficult to do with the standard electrophysiology-based approach using pharmacological inhibitors. Moreover, a similar approach could be used to identify additional acid-sensing mechanisms in other neural systems, including pain sensation/perception and fear/anxiety disorders. While the overall significance of these observations remains unclear (since the molecular underpinnings of pH sensitivity in brainstem 5-HT neurons remains unclear), further exploration of these and additional candidate genes and their functional validation is warranted.

## Author Contributions

MP and GM performed experiments, analyzed data, performed statistical analyses and contributed to manuscript preparation. PL analyzed sequencing data and performed statistical analyses. MH performed experiments and contributed to manuscript preparation.

## Conflict of Interest Statement

The authors declare that the research was conducted in the absence of any commercial or financial relationships that could be construed as a potential conflict of interest.

## Supplementary Material

TABLE 1 **Forward and reverse primer sequences for PCR**.
