# Supplementary material for: Identifying Candidate Genes that Underlie Cellular pH Sensitivity in Serotonin Neurons Using Transcriptomics: A Potential Role for Kir5.1 Channels
Source: Front Cell Neurosci. 2017 Feb 21;11:34. doi: 10.3389/fncel.2017.00034 (PMC5318415; doi:10.3389/fncel.2017.00034)
Supplement: TABLE 1 — Forward and reverse primer sequences for PCR. [file Table_1.PDF]

Table 1: Puissant et al.

Gene	Forward Primer	Reverse Primer
Tph2	GTAATACGACTCACTATAGGGCGCTACACGCAGAGCATTGAA	GAATTAACCCTCACTAAAGGGATCCATCCCAACTGCTTGTGT
Slc6a4	GTAATACGACTCACTATAGGGCGTCAAAACGTCTGGCAAGGT	GAATTAACCCTCACTAAAGGGATGACCACGATGAGCACAAA
Kcnj16	GACCATCCGCTTCAGCTATT	CACTGGCGTTACCAGGATTAT
Kcnj10	CCAAGGTCTATTACAGCCAGAC	CTGATGTAGCGGAAGCCATAG
Kcna2	CTACAGGAGCGCAGACTAATG	GAGAACCAGATGATGCAGAGAG
Kcnk3	CGGCTTCCGCAACGTCTAT	TTGTACCAGAGGCACGAGCA
Kcnk9	GACGTGCTGAGGAACACCTACTT	GTGTGCATTCCAGGAGGGA
